# Snapping up

**Published:** 1860-10

**Authors:** 


### SNAPPING UP.

MAD-DOGS and turtles are not the only snapping animals in the world. It is to be feared that most families are afflicted with one or more "snappers," who are wont to exercise their spitfire propensities especially at the table or around the family fireside. Addressing herself to her mother, Mary, with her eyes full of twinkle and fun, says: "I took a walk at ten o'clock this morning, and—" here John broke in. Now John was just at that age when a youth knows every thing under the sun, and more too; he never makes a mistake, is always positive that every thing he does, says or thinks, is just exactly so, and could not possibly be any other way. "Why, sister! how could you say it was ten o'clock? it was quarter-past ten at least!" One sample is enough. Every one of observation can, of his own knowledge, multiply cases indefinitely.

This unseemly habit is sometimes observed in families whose position and opportunities of association would lead to the supposition that every thing vulgar and uncourteous would be instinctively shunned. The person criticised, not having sense enough to pass over the boorishness, begins a defense: and before one is aware of it, the whole table or circle is silenced, and find themselves in the awkward position of listeners to a series of angry contradictions about a matter of no possible consequence to any one of the whole company in one sense, but of importance in another, as there is a certain disagreeableness about it which all feel more or less. What if a thing happened a minute or a month later or sooner? it is the general statement to which attention is directed. Contradictions and criticisms and corrections in general company are clownish; they are clear proof that, in almost every case, the person who assumes such an ungracious office is a boor of the first water, and is essentially deficient in that refinement and delicacy, which are inseparable from a cultivated mind and a taste for all that is beautiful, elegant and refined. A whole evening's enjoyment has been frequently marred, and all the company have gone home with a kind of blight upon the sensibilities, in consequence of a jar caused by the impertinent contradiction or correction of some unimportant fact in a narration. And as human health is promoted by a series of agreeable sensations, whatever interrupts that series in a single instance or for a single moment, is a legitimate object of demolition on the part of a JOURNAL OF HEALTH.

